# Analysis of microvascular and neurodegenerative complications of mild COVID-19

**DOI:** 10.1007/s00417-022-05623-8

**Published:** 2022-03-19

**Authors:** Zsofia Kolkedi, Adrienne Csutak, Eszter Szalai

**Affiliations:** Department of Ophthalmology, University of Pécs Medical School, Rákóczi u. 2, 7623 Pécs, Hungary

**Keywords:** COVID-19, In vivo confocal microscopy, Neuropathy, OCT angiography

## Abstract

**Purpose:**

To examine retinal and corneal neurodegenerative and retinal microvascular changes in patients after mild or asymptomatic COVID-19 disease compared to age-matched controls.

**Methods:**

Thirty-five **(**35) patients after PCR-proven SARS-CoV-2 infection and 28 age-matched controls were enrolled. Swept-source optical coherence tomography (OCT), OCT angiography, and in vivo corneal confocal microscopy were performed in both groups. Corneal subbasal nerve plexus was quantified. Vessel density for superficial (SCP) and deep capillary plexus (DCP) and structural OCT parameters were recorded.

**Results:**

Significantly lower nerve branch density (*P* = 0.0004), nerve fiber area (*P* = 0.0001), nerve fiber density (*P* = 0.0009), nerve fiber length (*P* < 0.0001), and total nerve branch density (*P* = 0.002) values were observed in patients after COVID-19 compared to healthy controls. VD of the temporal SCP was significantly different between the two groups (*P* = 0.019). No other SCP and DCP vessel density parameter differed significantly between the two groups.

**Conclusions:**

Our results suggest that peripheral neurodegenerative changes may occur even after mild or asymptomatic SARS-CoV-2 infection. No relevant microvascular changes were seen with OCT angiography and structural OCT parameters did not show any signs of optic neuropathy in post-COVID patients. In vivo confocal microscopy seems to be an important tool in monitoring peripheral neuropathy in patients after COVID-19.

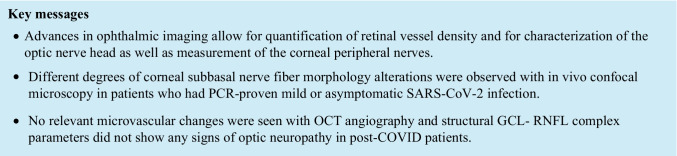



## Introduction

COVID-19 caused by severe acute respiratory syndrome coronavirus-2 (SARS-CoV-2) may cause a spectrum of symptoms ranging from fever and mild dyspnea to severe respiratory failure and some death [1]. The disease is known to have various ophthalmic manifestations including ocular surface involvement, uveitis, retinal damage, and neuroophthalmological complications [2, 3]. One-third of the previously hospitalized patients reported persistent COVID-19-related symptoms and half of them suffered from long-standing symptoms after hospital discharge [4].

Ophthalmologists have traditionally been able to directly observe and analyze the small vessels and cranial nerve II (optic nerve) on fundus examination. Recently, advances in imaging allow for quantification of vessel density and for the characterization of the optic nerve head as well as measurement of the corneal peripheral nerves (branches from the ophthalmic division of the trigeminal nerve). Thus, a comprehensive ophthalmic examination can provide general information on the vascular system and peripheral nerves in any systemic diseases.

General (fatigue, decreased exercise tolerance, breathlessness) and neuropsychological consequences (anxiety/depression, psychological distress) have been commonly reported in SARS survivors [5]. The microvascular and neurodegenerative complications of multisystemic diseases secondary to SARS-CoV-2 infection have been gaining more scientific attention [6, 7]. The purpose of this research was to examine retinal and corneal neurodegenerative and retinal microvascular changes with noninvasive clinical methods in patients who had mild COVID-19 disease.

## Material and methods

Sixty-three (63) subjects were prospectively enrolled from the Department of Ophthalmology, University of Pecs; 35 patients after PCR-proven SARS-CoV-2 infection with mild disease presentation and 28 age- and sex-matched controls were also enrolled. Control subjects had no past or current history of any systemic or ocular diseases and no participant in either group had a history of contact lens wear or intraocular surgery. In both study groups, research protocol included visual acuity measurement, slit lamp examination, intraocular pressure measurement, and anterior and posterior (dilated) segment imaging, with anterior (Anterion; Heidelberg Engineering, Heidelberg, Germany) and posterior segment (Topcon DRI OCT Triton Swept source OCT, Topcon, Japan) optical coherence tomography (OCT), OCT angiography (OCTA), and in vivo confocal microscopy (Heidelberg Retina Tomograph II Rostock Cornea Module; Heidelberg Engineering GmbH, Heidelberg, Germany). All participants provided written informed consent in accordance with the Declaration of Helsinki and the study was approved by the University of Pecs Institutional Ethical Review Board (number: 8672-PTE 2021).

All study subjects underwent in vivo imaging of the subbasal nerve fibers of the cornea. In brief, a drop of topical anesthetic (oxybuprocaine 0.4%) was applied to the subjects’ eye and viscous Vidisic gel (Bausch and Lomb, Berlin, Germany) was applied in a disposable sterile polymethylmethacrylate cap (Tomo-Cap; Heidelberg Engineering GmbH), which was placed over the objective lens. In both study groups, one eye was chosen randomly for the image analysis.

Three good-quality images of the subbasal nerve plexus were selected and analyzed by using ACCMetrics software V3 (University of Manchester, Manchester, UK) [8–13]. Corneal nerve fiber density (NFD), the number of nerve fibers/mm^2^; nerve branch density (NBD), the number of primary branch points on the main nerve fibers/mm^2^; nerve fiber length (NFL), the total length of nerves mm/mm^2^; nerve fiber total branch density (TBD), the total number of branch points/mm^2^; nerve fiber area (NFA), the total nerve fiber area mm^2^/mm^2^; and nerve fiber width (NFW), the average nerve fiber width mm/mm^2^, were calculated.

OCT and OCTA were performed for retinal structural and microvascular imaging, using swept-source OCT, DRI OCT Triton plus (Topcon, Tokyo, Japan). All OCT and OCTA images were acquired by well-trained examiner. OCTA imaging was performed with 3 mm × 3 mm volumetric scans centered at the fovea containing 320 × 320 A-scans. Low-quality OCTA images or presence of motion artifacts was excluded from the study. We used the automated layer segmentation for SCP and DCP using the built-in software segmentation algorithm (IMAGEnet 6 Version 1.26.16898, Topcon). The superficial capillary plexus (SCP) was delineated by 2.6 µm below internal limiting membrane to 15.6 µm below the junction between inner plexiform layer (IPL) and inner nuclear layer (INL), for deep capillary plexus (DCP), 15.6 µm below IPL/INL to 70.2 µm below IPL/INL. For each layer, the vessel density (VD) was automatically provided by IMAGEnet software. The quadrant VD (superior, inferior, nasal, and temporal) was analyzed using an Early Treatment Diabetic Retinopathy Study (ETDRS) grid containing the two inner rings. Foveal avascular zone (FAZ) was manually outlined by the same trained observer. Structural OCT was performed by using SMARTTrack HD Raster centered at the macula (6.0 × 6.0 mm) and 3D Disc program (6.0 × 6.0 mm) centered at the optic nerve head. From the automated segmentation OCT map, the retinal nerve fiber layer thickness (RNFL), the ganglion cell complex thickness (GCL + , GCL + +), and central choroidal thickness were evaluated. The analysis of the OCTA images was carefully reviewed by two independent examiners.

## Statistical analysis

Statistical analysis was performed using MedCalc Version 14.8.1 (MedCalc Software bvba, Ostend, Belgium) and IBM SPSS Statistics 25.0 (IBM Corp., Armonk, NY). Results are described as mean ± standard deviation (SD), and 95% confidence interval (CI) for the mean. The Kolmogorov–Smirnov test was used to test whether our data were normally distributed. For pairwise comparison, independent samples *t* test was performed. For bivariate correlation analysis, Pearson correlation test was applied. A *P* value ≤ 0.05 was considered statistically significant.

## Results

Thirty-five (35) eyes of 35 COVID-19 patients (18 males and 17 females, mean age: 43.3 ± 13.8 years, range 21–67 years) and 28 eyes of 28 age-matched healthy subjects (11 males and 17 females, mean age: 46.7 ± 17.6 years, range 20–67 years) were studied, and no significant difference was found between the two groups regarding age (*P* = 0.388) and sex (*P* = 0.345). The mean time between the first positive PCR test and the ophthalmic examination was 13.5 ± 6.1 weeks (between 2 and 26 weeks). Active and persistent symptoms included fever in 17 patients (49%), dysgeusia/anosmia in 13 patients (37%), fatigue in 11 cases (31%), coughing in 11 patients (31%), joint pain in 11 cases (31%), insomnia in 2 patients (6%), depression in 1 patient (3%), and tachycardia/palpitation in 1 patient (3%). Six patients (17%) reported ophthalmic symptoms during the infection, such as burning of the eye, foreign body sensation, orbital, and ocular pain. Three (3) subjects (9%) were completely asymptomatic during the disease. In the post-COVID group, 6 patients had well-controlled type 2 diabetes mellitus, 6 patients had hypertension (5 patients had both diabetes mellitus and hypertension), and 1 patient had a prior history of central retinal vein occlusion on the contralateral eye. No patient had any signs of retinopathy on the study eye.

**Fig. 1:**
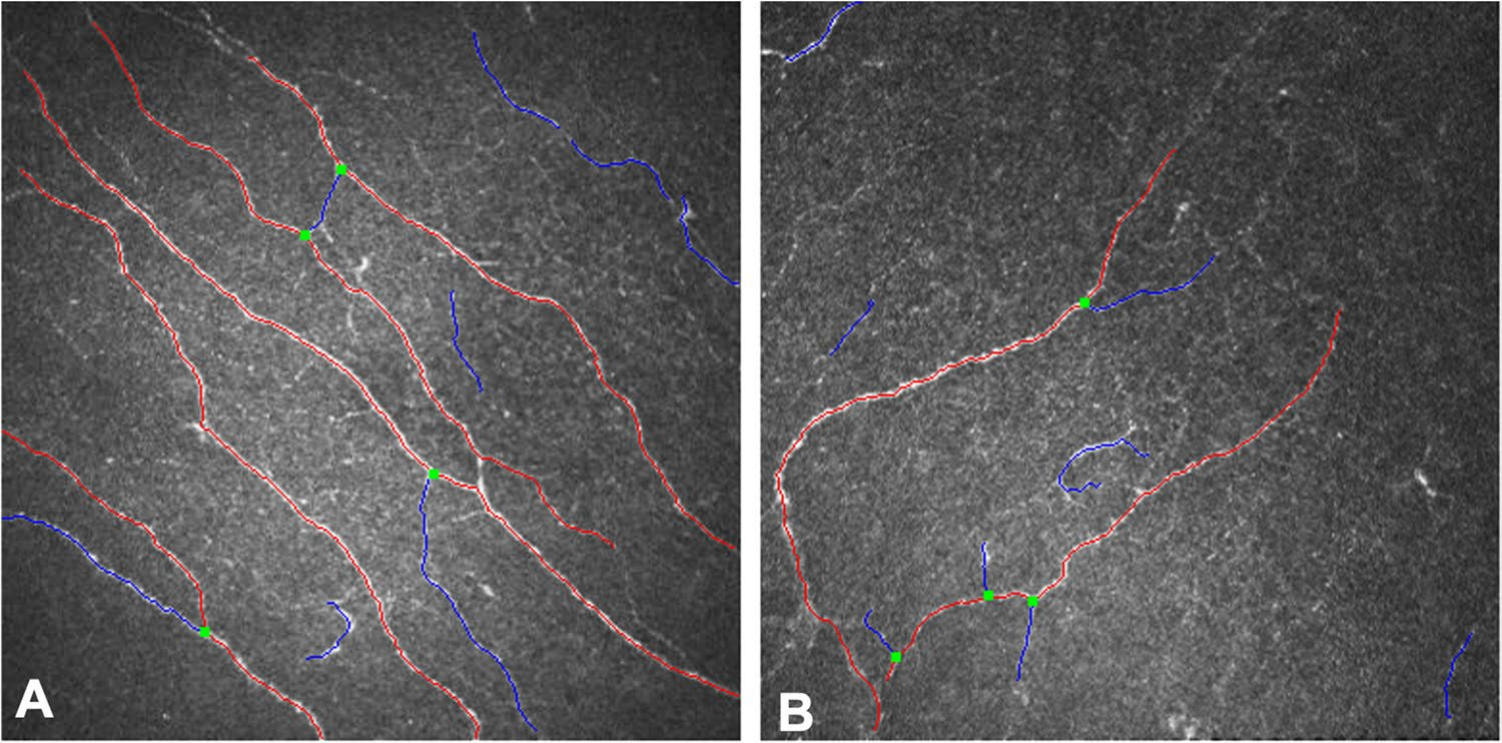
Analyzed image of the corneal subbasal nerve plexus using ACCMetrics software (red: fiber, blue: branch, green: branch point). **A** Normal nerve fiber morphology of a 69-year-old healthy subject; **B** decreased nerve fiber density and altered morphology of a 69-year-old patient after COVID-19

There was no statistically significant difference in ocular biometry measurements between the healthy and post-COVID subjects (Table 1). Significantly lower NBD (*P* = 0.0004), NFA (*P* = 0.0001), NFD (*P* = 0.0009), NFL (*P* < 0.0001), and TBD (*P* = 0.002) values were observed in patients after COVID-19 compared to healthy controls (Fig. 1) (Table 2). There was no significant difference in NFW between the two groups (*P* = 0.421).

**Table 1:** Ocular biometry in healthy subjects compared to patients after COVID-19

	Healthy subjects^§^	Patients after COVID-19^§^	P*
Anterior K1(D)	40.48 ± 1.763 (42.768–44.192)	42.828 ± 1.335 (42.354–43.301)	0.125
Anterior K2 (D)	44.203 ± 1.950 (43.415–44.991)	44.04 ± 2.117 (43.290–44.791)	0.737
Astigmatism (D)	0.74 ± 0.433 (0.565–0.915)	0.855 ± 0.4187 (0.706–1.003)	0.454
CCT (µm)	539.87 ± 29.579 (527.079–552.661)	545.848 ± 40.987 (531.315–560.382)	0.510
Internal ACD (mm)	2.785 ± 0.564 (2.557–3.013)	2.973 ± 0.440 (2.817–3.129)	0.148
WTW (mm)	11.826 ± 0.405 (11.658–11.993)	12.029 ± 0.383 (11.891–12.167)	0.062
LT (mm)	4.122 ± 0.318 (3.993–4.250)	4.208 ± 0.438 (4.048–4.369)	0.421
AL (mm)	23.43 ± 1.045 (22.923–23.767)	23.822 ± 1.043 (23.452–24.192)	0.087

*K*, keratometry; *CCT*, central corneal thickness; *ACD*, anterior chamber depth; *WTW*, white-to-white; *LT*, lens thickness; *AL*, axial length

^**§**^Mean ± standard deviation (95% confidence interval)

^*^Independent sample *t* test

**Table 2:** Corneal nerve fiber morphology of healthy subjects compared to patients after COVID-19

	Healthy subjects^§^	Patients after COVID-19^§^	P*
Nerve branch density (No/mm^2^)	23.63 ± 15.940 (17.449–29.810)	10.542 ± 11.350 (6.518–14.567)	0.0004
Nerve fiber area (mm^2^/mm^2^)	0.006 ± 0.002 (0.005–0.007)	0.004 ± 0.002 (0.003–0.004)	0.0001
Nerve fiber density (No/mm^2^)	18.57 ± 6.114 (16.200–20.941)	11.742 ± 8.649 (8.675–14.809)	0.0009
Nerve fiber length (mm/mm^2^	12.98 ± 3.141 (11.763–14.198)	8.605 ± 3.649 (7.311–9.899)	< 0.0001
Nerve fiber width (mm/mm^2^)	0.022 ± 0.0017 (0.0211–0.0224)	0.022 ± 0.002 (0.021–0.023)	0.421
Nerve fiber total branch density (No/mm^2^)	37.20 ± 20.415 (29.284–45.116)	21.937 ± 16.424 (16.113–27.760)	0.002

^**§**^Mean ± standard deviation (95% confidence interval)

^*^Independent samples *t* test

Central choroidal thickness was higher in the normal group but no statistically significant difference was found between the two groups (*P* = 0.101) (Table 3). VD of the temporal SCP was significantly different between the two groups (*P* = 0.019). No other SCP and DCP VD parameter differed significantly between the two groups (Table 3). RNFL thickness was higher in the normal subject but none of the RNFL-GCL complex parameters showed significant difference between normal and post-COVID subjects (Table 3).

**Table 3:** Optical coherence tomography (OCT) and OCT angiography parameters in healthy subjects compared to patients after COVID-19

	Healthy subjects^§^	Patients after COVID-19^§^	P*
Central choroidal thickness (µm)	304.769 ± 81.420 (271.883–337.656)	269.941 ± 79.458 (242.217–297.665)	0.101
VD of SCP CSF (%)	21.363 ± 5.248 (19.328–23.398)	22.931 ± 5.137 (21.139–24.723)	0.241
VD of SCP superior (%)	51.32 ± 2.357 (50.406–52.234)	50.546 ± 3.010 (49.496–51.596)	0.272
VD of SCP temporal (%)	48.526 ± 2.666 (47.492–49.560)	47.166 ± 1.761 (46.551–47.780)	0.019
VD of SCP inferior (%)	50.916 ± 3.312 (49.632–52.201)	50.714 ± 2.649 (49.775–51.654)	0.792
VD of SCP nasal (%)	47.154 ± 2.899 (46.030–48.278)	46.254 ± 1.974 (45.565–46.943)	0.153
SCP FAZ area (µm)	267.907 ± 83.553 (234.854–300.959)	252.822 ± 199.750 (210.361–295.284)	0.582
VD of DCP CSF (%)	19.065 ± 3.688 (17.575–20.555)	18.764 ± 5.437 (16.836–20.691)	0.810
VD of DCP superior (%)	52.071 ± 3.682 (50.644–53.499)	53.006 ± 3.688 (51.719–54,293)	0.324
VD of DCP temporal (%)	47.941 ± 3.768 (46.480–49.403)	47.115 ± 2.420 (46.271–47.959)	0.300
VD of DCP inferior (%)	52.011 ± 2.814 (50.920–53.102)	52.943 ± 3.253 (51.808–54.078)	0.444
VD of DCP nasal (%)	48.594 ± 3.096 (47.393–49.794)	47.776 ± 3.815 (46.445–49.107)	0.365
GCL + + CSF (µm)	52.481 ± 8.116 (49.271–55.692)	61.353 ± 25.830 (52.340–70.366)	0.091
GCL + + inner ring (µm)	116.454 ± 7.325 (113.556–119.351)	114.992 ± 12.067 (110.782–119.203)	0.582
GCL + + outer ring (µm)	108.731 ± 6.822 (106.033–111.430)	117.809 ± 65.197 (95.061–140.557)	0.475
GCL + CSF (µm)	52.481 ± 8.116 (49.271–55.692)	61.353 ± 25.830 (52.340–70.366)	0.091
GCL + inner ring (µm)	90.417 ± 6.189 (87.968–92.865)	100.338 ± 40.810 (86.099–114.578)	0.216
GCL + outer ring (µm)	67.778 ± 6.402 (65.245–70.310)	74.279 ± 34.012 (62.412–86.147)	0.332
RNFL total thickness (µm)	108.269 ± 10.850 (103.887–112.652)	103.235 ± 12.524 (98.865–107.605)	0.108

*VD*, vessel density; *SCP*, superficial capillary plexus; *DCP*, deep capillary plexus; *FAZ*, foveal avascular zone; *GCL*, ganglion cell layer; *RNFL*, retinal nerve fiber layer; *CSF*, thickness within central 1 mm; inner ring, thickness within central 3 mm; outer ring, thickness within central 6 mm

^**§**^Mean ± standard deviation (95% confidence interval)

^*^Independent samples *t* test

After excluding patients with metabolic diseases from the post-COVID group, a significant decrease was observed in NBD (*P* = 0.0002), NFA (*P* = 0.0001), NFD (*P* = 0.0001), NFL (*P* < 0.0001), and TBD (*P* = 0.0023). NFW did not differ significantly between the two groups (*P* = 0.206). No statistically significant difference was observed in VD parameters of the SCP and DCP (*P* = 0.102–0.894); only a borderline significant decrease was found in the temporal SCP VD (*P* = 0.051) in the post-COVID group.

Statistically significant correlation was found between NFW and nasal VD in SCP (*r* = 0.618, *P* = 0.0001) and DCP (*r* = 0.679, *P* = 0.0001) in the normal group. There was a significant inverse correlation between NFW and nasal VD in DCP (*r* =  − 0.391, *P* = 0.027) in the post-COVID group. No other statistically significant correlation was found between the corneal nerve fiber morphology and OCT angiography parameters. The time between the first positive PCR and ophthalmic examination did not show significant correlation with any of the measured parameters (*P* > 0.05).

## Discussion

It has been reported that many patients after the acute phase of the SARS-CoV-2 infection have persistent neurologic and autonomic symptoms. This has recently been referred to as “long-haul” COVID [14]. Objective testing and comprehensive examinations of the nonspecific symptoms in these patients have typically been inconclusive. Nath A. proposed the potential pathophysiologic mechanisms for long-haul COVID including residual damage from the infection, persistent virus replication, constant immune activation, and comorbidities [14]. Autoimmune events, inflammatory response or direct damage, and degenerative changes of different organs could explain some of the symptoms of acute and long-haul disease [14]. The inner retina is a neuronal tissue and may also be injured by SARS-CoV-2 as has been reported for the central nervous system [15]. In 2002–2003, SARS-CoV was found to be associated with occasional disease of the central and peripheral nervous system [16].

It has been shown that in vivo confocal microscopy could reveal early corneal microstructural and subbasal nerve fiber changes in patients with metabolic diseases before the development of ophthalmoscopic changes; thus, it might be an applicable tool for peripheral neuropathy screening [17]. In the post-COVID group, we identified decreased number of primary branch points on the main nerve fibers (NBD) and lower total number of branch points confirming distal loss of nerve branches (NTBD) with normal nerve fiber width (NFW). There was a significant reduction in the number of nerve fibers (NFD) reflecting the more proximal nerves after COVID-19. Consequently, a significantly decreased total nerve fiber area (NFA) was seen in the post-COVID group. Retinal neurodegeneration was also examined. Quantification of neuronal loss was obtained by studying the GCL-RNFL complex. RNFL thickness did not show significant difference between the healthy and post-COVID subjects. Previous authors observed no difference between post-COVID and normal subjects in structural parameters of GCL-RNFL complex; however, they assumed microvascular peripapillary involvement in SARS-CoV-2 infection [18, 19]. Due to the possible confounding effect of co-existing systemic diseases (e.g., diabetes mellitus and/or hypertension) in the study population, we performed a subset analysis by excluding patients with metabolic disorders. It did not alter our final results, so changes were not attributable to either diabetes or hypertension in the post-COVID group.

SARS-CoV-2 coronavirus recognizes and uses angiotensin-converting enzyme 2 (ACE-2) receptor to enter into different cells [20]. ACE-2 receptor is expressed in the retina and choroid and on different cell types such as the Müller cells, ganglion cells, photoreceptor cells, and the retinal vascular endothelial cells [20, 21]. Thus, it is involved in the pathogenesis of systemic vascular diseases including diabetic and hypertensive retinopathy [22]. In addition, SARS-CoV-2 viral RNA was detected in the retina in 21% of deceased COVID-19 patients [23].

We also studied microvascular changes in the retinal capillary network and choriocapillaris after SARS-CoV-2 infection. In general, we did not observe significant difference in vascular density either in SCP or in DCP when compared to the healthy group. Only VD of the temporal SCP decreased significantly in patients after COVID-19. In healthy subjects, VD of the SCP showed significant decrease from the foveal center, with a 51% loss on the temporal side measured with swept-source OCTA [24]. In diabetic patients, VD in the temporal perifoveal region was the most sensitive for early detection of retinopathy which was explained by the anatomic arrangement of the retinal vasculature [25]. Abrishami et al. evaluated 31 patients 2 weeks after recovery from COVID-19 and detected a statistically significant lower foveal and parafoveal vascular density both in SCP and DCP compared to a retrospective healthy cohort [26]. They presumed it to be explained by the higher prevalence of comorbidities (immunological diseases, obesity, diabetes mellitus, and cardiovascular diseases) [18]. Similar findings, however, were also found with OCTA in a cohort of young post-COVID patients without pre-existing systemic conditions [26]. Microvascular injury and thrombotic events were reported in patients with severe COVID-19 which draw the attention to the importance of qualifying and quantifying retinal microvascular involvement with OCTA [26].

In our study, different degrees of corneal subbasal nerve fiber morphology alterations could be detected with in vivo confocal microscopy in patients who had PCR-proven mild or asymptomatic SARS-CoV-2 infection. No relevant microvascular changes were seen with OCT angiography and structural GCL-RNFL complex parameters did not show any signs of optic neuropathy in post-COVID patients. Our results suggest that peripheral neurodegenerative changes may occur even after mild or asymptomatic SARS-CoV-2 infection. In vivo confocal microscopy seems to be an important tool in monitoring peripheral neuropathy in patients after COVID-19. Further investigations are needed to examine microvascular and neurodegenerative changes in patients after severe SARS-CoV-2 infections and to monitor the regeneration process of the affected peripheral nerves.

## Data Availability

Not applicable.
